# Candidate promoter-associated factors of *BrAGPS1* identified by yeast one-hybrid screening in Chinese cabbage during *Plasmodiophora brassicae* infection

**DOI:** 10.3389/fpls.2026.1916221

**Published:** 2026-09-15

**Authors:** Xiaohui Wang, Taining Ye, Yixuan Wu, Ying Li, Qiuhua Yan, Jing Jiang, Yue Liang, Wenxing Pang

**Affiliations:** 1 College of Horticulture, Shenyang Agricultural University, Shenyang, China; 2 College of Plant Protection, Shenyang Agricultural University, Shenyang, China

**Keywords:** BrAGPS1, Brassica rapa, *Plasmodiophora brassicae*, starch biosynthesis, yeast one-hybrid

## Abstract

Clubroot disease is caused by the obligate biotrophic protist *Plasmodiophora brassicae* and severely limits Chinese cabbage (*Brassica rapa* subsp. *pekinensis*) production. Infection is accompanied by extensive reprogramming of host carbon metabolism and starch accumulation in enlarged roots. ADP-glucose pyrophosphorylase (AGPase) catalyzes the first committed step of starch biosynthesis, but the upstream regulation of the Chinese cabbage small-subunit gene *BrAGPS1* remains poorly understood. We cloned the promoter regions of *BrAGPS1a* (*BraA07g007740.3.5C*) and *BrAGPS1b* (*BraA01g014740.3.5C*) from the inbred line ‘Chiifu-401-42’. PlantCARE analysis predicted core promoter motifs and putative cis-regulatory elements associated with hormone, stress, and light responses. A root cDNA library prepared from 1, 3, and 5 weeks post-inoculation (wpi) was screened by yeast one-hybrid (Y1H) using selected promoter fragments as baits to recover candidate promoter-associated proteins. The screen yielded 76 nonredundant library hits, 15 of which were annotated as proteins with potential transcriptional, DNA-binding, histone-binding, or chromatin-associated functions. Integration with an RNA-sequencing (RNA-seq) time course at 1, 2, 3, and 5 wpi identified 28 Y1H-derived genes that were differentially expressed at one or more time points. Five candidates—a calcium-dependent protein kinase (CPK), a basic leucine zipper (bZIP) transcription factor, a three-high-mobility-group-box (3×HMG-box) protein, and two Alfin1-like plant-homeodomain (PHD-domain) proteins—were selected on the basis of functional annotation and infection-responsive expression. Quantitative real-time PCR (qRT-PCR) confirmed distinct treatment- and time-dependent expression patterns but did not test DNA binding or regulatory function. This study provides a prioritized set of *BrAGPS1* promoter-associated candidates and testable hypotheses for investigating the transcriptional control of starch metabolism during clubroot development. One-to-one Y1H back-assays, electrophoretic mobility shift assays, promoter transactivation tests, and genetic analyses are required to establish direct binding, regulatory direction, and biological function.

## 1 Introduction

Clubroot is a globally important soil-borne disease of cruciferous crops caused by *Plasmodiophora brassicae* (Dixon, 2009). In China, it was first reported in Taiwan and Fujian in the early 1920s (Chai et al., 2014) and has progressively spread nationwide since the 1950s (Howard et al., 2010). In recent years, with the intensification and regional expansion of cruciferous cultivation, clubroot has extended into inland regions of northern and western China (Zheng et al., 2019). Almost all cultivated crucifers, including Chinese cabbage, radish, rapeseed, and cabbage, are susceptible to *P. brassicae* (Yang et al., 2020). As China is a major global producer of cruciferous vegetables (Zhu et al., 2023), the rapid spread and persistence of clubroot pose a serious threat to vegetable production and food security.

The resting spores of *P. brassicae* survive for many years in soil and tolerate adverse environmental conditions, making clubroot extremely difficult to manage (Kageyama and Asano, 2009). Infection induces abnormal proliferation of root tissues and the formation of galls of various sizes on primary and lateral roots. These galls act as strong nutrient sinks, consuming large amounts of assimilates while impairing root water and nutrient uptake (Voorrips, 1995). Consequently, infected plants exhibit reduced growth and development, stunting, weak plant architecture, and progressive yellowing and wilting of basal leaves. Under current production conditions, green and integrated management strategies have become essential. Approaches combining physical and chemical measures, crop rotation, soil pH adjustment, and the deployment of clubroot-resistant cultivars have shown promise in reducing disease incidence and severity (Henning et al., 2022; Ernst et al., 2019; McDonald et al., 2020; Peng et al., 2014).

Clubroot development is accompanied by extensive metabolic reprogramming, including marked starch accumulation in infected root tissues. This accumulation is consistently associated with gall development, but whether the stored carbon directly benefits the pathogen, represents a host response, or reflects altered sink metabolism remains unresolved. Clarifying the regulation of host starch-biosynthetic genes is therefore relevant to understanding the infected-root metabolic state without presuming a direct nutritional benefit to *P. brassicae*.

Starch, the major end product of photosynthesis in source tissues and a key carbon reserve in sink organs, consists of two glucose polymers, amylose and amylopectin (Liu and Cai, 2011). The immediate precursor for starch synthesis is ADP-glucose (ADPG), produced from glucose-1-phosphate and ATP by ADP-glucose pyrophosphorylase (AGPase). This reaction constitutes the first committed and rate-limiting step in starch biosynthesis (Tetlow et al., 2003; Bowsher et al., 2007; Villand and Kleczkowski, 1994). In rice, AGPase is a heterotetramer composed of two small subunits (AGPS1 and AGPS2) and two large subunits (AGPL1–AGPL4) (Ohdan et al., 2005; Lee et al., 2007). The small subunit (AGPS) harbors both the catalytic and regulatory sites; it is activated by 3-phosphoglycerate and inhibited by inorganic phosphate (Sikka et al., 2001). AGPS1 produces two splice variants: AGPS1b, which contains a transit peptide and is plastid-localized, and AGPS1a, which lacks the transit peptide and is cytosolic (Ohdan et al., 2005). In *Brassica* species, genome-wide analyses have identified eight AGPase-encoding genes in *B. rapa*, including homologs of *AGPS1* (Ma et al., 2022). Our previous work demonstrated that *BrAGPS* genes are significantly upregulated in Chinese cabbage roots following *P. brassicae* inoculation, suggesting that AGPase-mediated starch synthesis is actively reprogrammed during clubroot infection (Ma et al., 2022).

Transcriptome sequencing has greatly facilitated the dissection of host–pathogen interactions in clubroot. RNA-seq analyses of Chinese cabbage roots infected by *P. brassicae* have revealed extensive transcriptional reprogramming in genes related to disease resistance, stress response, and primary and secondary metabolism (Wang et al., 2024). Comparative transcriptomics of resistant and susceptible Chinese cabbage varieties have further implicated auxin signaling, disease-resistance proteins, oxidative stress-related genes, and WRKY and MYB transcription factors (TFs) in clubroot resistance regulation (Yuan et al., 2021). These studies provide a framework for elucidating the molecular mechanisms underlying *P. brassicae* infection and host defense.

Yeast one-hybrid (Y1H) assays detect proteins that enable reporter activation through association with a bait DNA sequence and are widely used to identify candidate protein-DNA interactions (Cao et al., 2021; Fuxman Bass et al., 2016). Because library screens can recover false positives and noncanonical proteins, individual hits require one-to-one retesting and orthogonal validation before direct binding to a specific cis-element can be concluded. In plant studies, Y1H has been used to nominate upstream regulators of defense-, hormone-, and stress-responsive genes (Huan et al., 2017; Liao et al., 2015).

Our previous analysis of *Brassica rapa* roots infected by *P. brassicae* identified *BrAGPS1* as an infection-responsive starch-biosynthesis gene, motivating investigation of its upstream regulation. In the present study, PlantCARE prediction was performed first and was used to guide partitioning of the *BrAGPS1a* and *BrAGPS1b* promoters into candidate bait regions. Y1H library screening then identified proteins associated with the tested promoter fragments. The design does not, by itself, assign any recovered protein to an individual predicted cis-element. Candidates were subsequently ranked by sequence annotation and their expression responses in an independent infection time course.

Accordingly, this work aimed to (i) characterize predicted cis-acting elements in the *BrAGPS1a* and *BrAGPS1b* promoters, (ii) identify candidate genes associated with selected promoter fragments by Y1H library screening, and (iii) evaluate the infection-responsive expression of prioritized candidates. The resulting candidate set is intended to guide direct binding and functional studies rather than to establish a validated regulatory network or clubroot-resistance mechanism.

## 2 Materials and methods

### 2.1 Plant material and inoculation procedure

Seeds of the Chinese cabbage (*Brassica rapa* ssp. pekinensis) inbred line ‘Chiifu-401-42’ were kindly provided by Professor Lim Yong Pyo (Chungnam National University, South Korea). The *P. brassicae* isolate used in this study was Pb4, identified according to the Sinitic clubroot differential (SCD) host system (Pang et al., 2020). Phenotypic verification confirmed that ‘Chiifu-401-42’ is highly susceptible to Pb4; therefore, this inbred line was selected as the susceptible host material for exploring the interaction between host starch metabolism and pathogen infection. Clubroot-infected roots preserved at -20 °C in the Molecular Biology Laboratory of Shenyang Agricultural University were thawed on ice. Approximately 5 g of diseased root tissue was weighed, cut into small pieces, transferred to a glass beaker, and homogenized in distilled water using a high-speed homogenizer to obtain a root slurry. The homogenate was filtered through eight layers of sterile gauze to remove debris, and the filtrate was collected as a spore suspension. This suspension was diluted with distilled water to prepare a resting spore suspension. Spore concentration was determined with a hemocytometer under a light microscope and adjusted to 1 × 10^7^ resting spores·mL^-1^.

Chinese cabbage seeds were sown and grown under standard greenhouse conditions (25 ± 2 °C day/20 ± 2 °C night, 16 h light/8 h dark photoperiod, relative humidity 60-70%) until the two-true-leaf plus one-heart stage. At this stage, 3 mL of the prepared resting spore suspension was injected at the stem base of each seedling using a sterile syringe. Samples were harvested at 1, 3, and 5 weeks post-inoculation (wpi). Shoots and roots were carefully washed with tap water followed by distilled water. Roots were excised with a sterile blade, wrapped in aluminum foil, and immediately frozen in liquid nitrogen; the same procedure was applied to the above-ground tissues. All samples were stored at −80 °C until further use. Mock-treated plants received the same 3 mL volume of sterile distilled water without resting spores.

### 2.2 Construction of the yeast cDNA library

Total RNA was extracted separately from Chinese cabbage roots collected at 1, 3, and 5 wpi using TRIzol reagent. Equal RNA masses from the three infected-root time points were pooled before first-strand cDNA synthesis. After second-strand synthesis, the resulting double-stranded cDNA was subjected to Gateway BP recombination and transformed into *Escherichia coli* to generate the primary cDNA library.

To estimate the primary library titer, 10 μL of the transformed *E. coli* culture was diluted 100-fold with super optimal broth with catabolite repression (SOC) medium, and 50 μL of the diluted suspension was spread onto Luria–Bertani (LB) plates containing 50 μg·mL^-1^ kanamycin. Plates were incubated overnight at 37 °C in an inverted position. After colony formation, the number of colonies was recorded, and the library titer was calculated as:


$$\text{CFU}\cdot {\text{mL}}^{-1}\;=\left(\text{colony number}/50\;\mu \text{L}\right)\times 100\times 1\times 10^{3}\;\mu \text{L}$$

$$\text{Total library size}\left(\text{CFU}\right)=\text{CFU}\cdot {\text{mL}}^{-1}\times \text{total library volume }\left(\text{mL}\right).$$

Twenty-four single colonies were randomly selected for colony PCR to determine the recombination rate and insert size distribution. After primary library validation, cDNA inserts were transferred into the pGADT7 vector via Gateway LR recombination. The recombinant plasmids were transformed into *E. coli* to obtain the secondary cDNA library. As above, 10 μL of transformed culture was diluted 100-fold with SOC, and 50 μL was plated on LB plates containing ampicillin. After overnight incubation at 37 °C, colonies were counted, and 24 colonies were randomly picked for insert verification by colony PCR.

### 2.3 Genomic DNA extraction, PCR amplification, and vector construction

Total genomic DNA was extracted from plant tissues using the cetyltrimethylammonium bromide (CTAB) method. Primers were designed with the BioRun online tool (https://www.biorun.com/tools/) and synthesized by Suzhou Genewiz Biotechnology Co., Ltd. PCR reactions were performed using 2× SanTaq PCR Master Mix, and products were resolved on 1% (w/v) agarose gels. Single bands of the expected size were excised, purified with the SanPrep Column DNA Gel Extraction Kit (Sangon Biotech), and ligated into pMD19-T according to the manufacturer’s protocol. Positive transformants were identified by colony PCR and Sanger sequencing. Confirmed clones were cultured overnight, and plasmid DNA was prepared using standard procedures described by Green and Sambrook (2012).

### 2.4 Promoter retrieval, cis-element analysis, bait-vector construction, and self-activation testing

The *BrAGPS1a* and *BrAGPS1b* gene models were located in the *Brassica rapa* Chiifu v3.5 assembly at A07:7,817,171–7,819,610 and A01:7,806,501–7,810,720, respectively (1-based coordinates). Putative 2,000-nt upstream promoter sequences were retrieved from the Brassicaceae Database (BRAD) genome resource and analyzed with PlantCARE.

Promoter fragments were cloned into the bait vector by restriction digestion and ligation. The recombinant bait constructs were transformed into yeast Y1HGold competent cells, and correct integration into the yeast genome was confirmed by colony PCR using pAbAi vector-specific primers paired with promoter-specific primers, followed by Sanger sequencing of the amplicons to verify junction integrity. To determine the minimal aureobasidin A (AbA) concentration required to suppress self-activation, single colonies of correctly integrated bait strains grown on synthetic dropout medium lacking uracil (SD/−Ura) plates were resuspended in 0.9% NaCl and adjusted to an optical density at 600 nm (OD_600_) of approximately 0.002. Then, 100 μL of the suspension was spread onto SD/−Ura plates supplemented with 0, 100, 150, 200, or 400 ng·mL^-1^ AbA. Plates were incubated at 30 °C for 2–3 days. Based on colony growth, an appropriate AbA concentration was selected for subsequent library screening.

### 2.5 Yeast one-hybrid library screening

Yeast competent cells harboring the bait constructs were prepared according to the Matchmaker^®^ Gold Yeast One-Hybrid Library Screening System user manual (Clontech/TaKaRa, Cat. No. 630491). For each transformation, 10 μg of cDNA library plasmid and 50 μL of pre-denatured carrier DNA were added to the competent cells and gently mixed. Then, 2.5 mL of 1× polyethylene glycol/lithium acetate (PEG/LiAc) solution was added, mixed thoroughly, and the mixture was incubated at 30 °C for 45 min, with gentle inversion every 15 min. Next, 160 μL of dimethyl sulfoxide (DMSO) was added, mixed gently, and cells were subjected to heat shock at 42 °C for 20 min, mixing every 10 min. Cells were collected by centrifugation at 700 × g for 5 min, resuspended in 3 mL of yeast extract–peptone–dextrose (YPD) Plus medium, and shaken at 30 °C for 90 min. After a second centrifugation (700 × g, 5 min), the pellet was resuspended in 0.9% NaCl to a final volume of 15 mL.

For titer determination, 100-µL aliquots of the 1/10, 1/100, and 1/1,000 dilutions were spread on 100-mm synthetic dropout medium lacking leucine and supplemented with AbA (SD/−Leu/AbA) plates. 150 µL was spread on each of 150-mm selective plates per bait transformation for the two screens (40 plates in total). Plates were incubated upside down at 30 °C for 3–5 days. Colonies >1 mm were picked, restreaked, and analyzed by colony PCR with universal pGADT7 primers. p53-AbAi plus pGADT7-Rec-p53 was the positive control, and p53-AbAi plus empty pGADT7 was the negative control. Single-band products were eligible for sequencing; when several products had indistinguishable apparent sizes, only the strongest representative was sequenced, a step that may have under-sampled distinct inserts of similar length. Sequence identities were deduplicated after Sanger sequencing.

Recovered insert sequences were quality-trimmed and queried against the National Center for Biotechnology Information (NCBI) and BRAD databases using the Basic Local Alignment Search Tool (BLAST). Candidate annotations were assigned from the best-supported v3.5 gene model, conserved-domain evidence, and published functional information.

### 2.6 RNA-seq library preparation, sequencing, and differential gene expression analysis

Root samples were collected at 1, 2, 3, and 5 wpi after inoculation with *P. brassicae* isolate Pb4 or sterile distilled water (mock control). Three independent biological replicates were collected for each treatment and time point; each replicate comprised roots pooled from 20 plants, giving 24 RNA-seq libraries (2 treatments × 4 time points × 3 replicates). In contrast, equal RNA masses from inoculated roots at 1, 3, and 5 wpi were pooled only for construction of the Y1H cDNA library; the 2-wpi samples were used exclusively for RNA-seq. Novogene Co., Ltd. performed RNA quality control and 150-bp paired-end sequencing on an Illumina NovaSeq 6000. Clean reads were aligned to the *Brassica rapa* cv. Chiifu v3.5 reference genome with HISAT2 v2.2.1. Raw integer counts were analyzed with DESeq2 v1.34.0 using its median-of-ratios normalization; fragments per kilobase of transcript per million mapped reads (FPKM) were used only for visualization and heatmap clustering. The model included time, treatment, and their interaction; genes with |log_2_FC| ≥ 1 and Benjamini-Hochberg-adjusted *p* ≤ 0.05 were considered differentially expressed.

### 2.7 Expression analysis of candidate genes by quantitative real-time PCR (qRT-PCR)

Root samples from inoculated and mock-control plants were collected at 1, 3, and 5 wpi. Total RNA was isolated, reverse-transcribed, and analyzed by quantitative real-time PCR (qRT-PCR). Candidate-gene primers were designed from coding sequences with BioRun (https://www.biorun.com/tools/101.html). *BrActin* was used as the internal reference gene. The reference primers were 5′-ATCTACGAGGGTTATGCT-3′ (forward) and 5′-CCACTGAGGACGATGTTT-3′ (reverse) (Xin et al., 2022). All target and reference primer sequences are listed in **Supplementary Table S2**.

### 2.8 Statistical analysis

Experiments included three independent biological replicates unless otherwise stated; three technical qRT-PCR reactions were run for each biological replicate. Technical cycle-threshold (Ct) values were averaged first, and biological replicates were used as the independent observations for statistical analysis. Relative transcript levels were calculated by the 2^-ΔΔCt^ method and are presented as mean ± standard deviation (SD). Analyses were performed in SPSS 20.0. One-way analysis of variance followed by Duncan’s multiple range test was used for comparisons involving more than two groups. Inoculated and mock-control samples at an individual time point were compared with a two-sided Student’s t-test. Thresholds were p< 0.05, p< 0.01, and p< 0.001. Graphs were prepared in Origin 9.1.

## 3 Results

### 3.1 Construction of yeast cDNA library

Chinese cabbage inbred line ‘Chiifu-401-42’ seedlings were inoculated with *P. brassicae* using the stem-base injection method. Plants were sampled at 1, 3, and 5 wpi and compared with non-inoculated controls (CK). In the *P. brassicae*-inoculated group (Pb), no visible root galls were observed at 1 wpi, whereas visible galls were evident at 3 and 5 wpi (**Figure 1A**). These observations confirmed successful infection and indicated that the collected samples were suitable for subsequent Y1H cDNA library construction.

**Figure 1:**
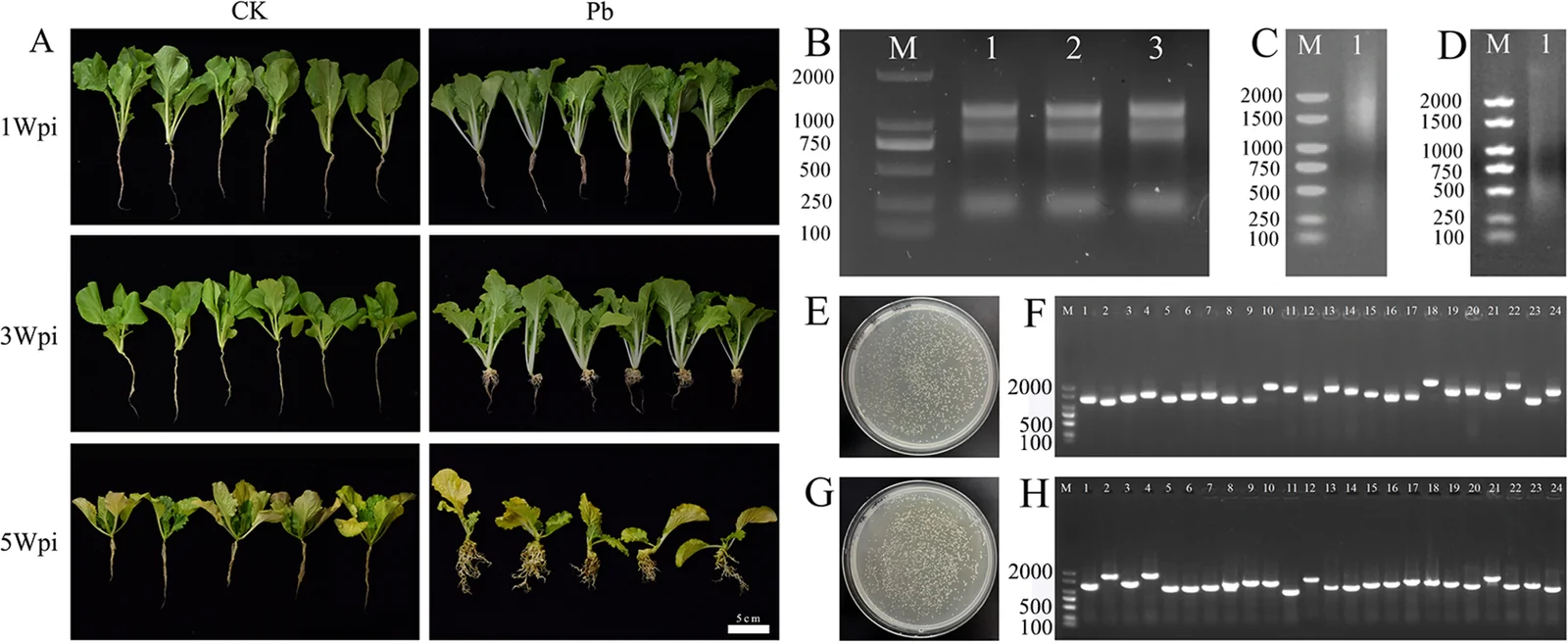
Construction and quality assessment of the Chinese cabbage root cDNA library used for yeast one-hybrid (Y1H) screening. **(A)** Phenotypes of Chinese cabbage inbred line ‘Chiifu-401-42’ at 1, 3, and 5 wpi after mock treatment (CK) or inoculation with *P. brassicae* (Pb). **(B)** Total root RNA: M, DL2000 DNA marker; lanes 1–3, 1, 3, and 5 wpi, respectively. **(C)** mRNA isolated from pooled total RNA: M, DL2000 DNA marker; lane 1, mRNA. **(D)** Double-stranded cDNA synthesized from root mRNA: M, DL2000 DNA marker. **(E–H)** Library capacity and insert-size assessments. **(E)** Colonies on kanamycin-selective LB medium for primary-library titer estimation. **(F)** Colony PCR of 24 primary-library clones. **(G)** Colonies on ampicillin-selective LB medium for secondary-library titer estimation. **(H)** Colony PCR of 24 secondary-library clones. The 5-cm scale bar applies to all images in panel **(A)**.

Total RNA was extracted from roots at 1, 3, and 5 wpi (**Figure 1B**). Agarose gel electrophoresis showed three distinct rRNA bands with an approximate 25S:18S ratio of 2:1, indicating good RNA integrity without obvious degradation. After pooling the three RNA samples, the concentration was 980.9 ng·μL^-1^, with an A_260_/A_280_ ratio of 2.16 and an A_260_/A_230_ ratio of 2.32, demonstrating high purity and absence of phenol or other organic contaminants. The isolated mRNA displayed a continuous smear of appropriate size (**Figure 1C**), consistent with the requirements for library construction. Double-stranded cDNA (ds cDNA) synthesized from the RNA was examined by agarose gel electrophoresis; the ds cDNA showed a dispersed banding pattern (**Figure 1D**), indicating that the synthesized cDNA was of sufficient quality for library construction.

A total of 1,300 colonies grew on the kanamycin-selective LB plate used for primary library titer determination (**Figure 1E**). Based on the plating volume and dilution factor, the primary library titer and total capacity were calculated to be 2.60 × 10^6^ CFU·mL^-1^, with a total capacity of 1.04 × 10^7^ CFU.

Twenty-four single colonies from the primary library were randomly selected for colony PCR (**Figure 1F**). All lanes displayed clear single bands. Comparison with the DL2000 marker indicated that insert sizes were generally >1,000 bp, and the recombination efficiency reached 100%. These results demonstrate that the primary library had high insert integrity and sufficient capacity for further processing.

For the secondary library, 1,500 colonies were obtained on the ampicillin-selective LB plate (**Figure 1G**). The calculated titer and capacity were:

The secondary library titer was calculated to be 3.00 × 10^6^ CFU·mL^-1^, with a total capacity of 1.20 × 10^7^ CFU. Again, 24 single colonies were randomly chosen for colony PCR (**Figure 1H**). All lanes showed bright, single bands, and the majority of inserts were >1,000 bp with a recombination efficiency of 100%. Collectively, these data indicate that the constructed cDNA library possessed high quality, large capacity, and suitable insert sizes to support subsequent Y1H screening.

### 3.2 Promoter motif analysis, cloning of *BrAGPS1a* and *BrAGPS1b* fragments, and bait-vector construction

The *BrAGPS1a* and *BrAGPS1b* gene models were identified in the *Brassica rapa* Chiifu v3.5 assembly, and putative upstream regions were retrieved from BRAD. PlantCARE analysis predicted multiple hormone-, stress-, and light-responsive motifs, including ABRE, G-box, MYB, MYC, and light-responsive elements (**Figure 2**). These are computationally predicted motif occurrences and should not be interpreted as experimentally validated cis-regulatory elements.

**Figure 2:**
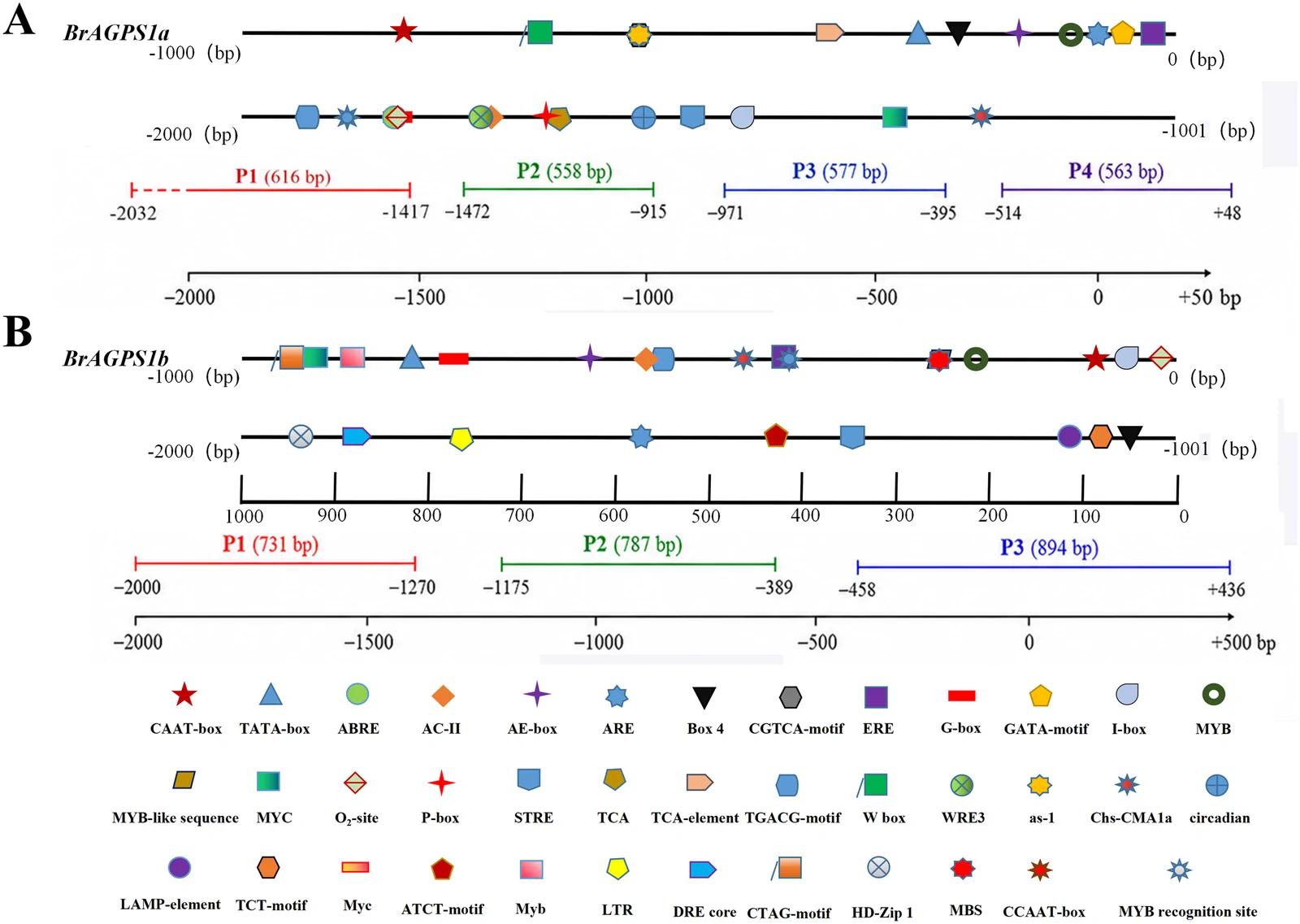
Predicted cis-regulatory motifs and Y1H bait-fragment boundaries in the *BrAGPS1a* **(A)** and *BrAGPS1b* **(B)** promoter regions. Motifs were predicted with PlantCARE; colored symbols denote motif classes, and the lower diagrams show the currently reported fragment boundaries relative to the stated coordinate origin. ABRE, abscisic-acid-responsive element; ARE, anaerobic-responsive element; DRE, dehydration-responsive element; LTR, low-temperature-responsive element; MBS, MYB-binding site; MeJA, methyl jasmonate; STRE, stress-responsive element.

**Figure 2** also shows the current bait-fragment boundaries: *BrAGPS1a* P1, −2,032 to −1,417 (616 bp); P2, −1,472 to −915 (558 bp); P3, −971 to −395 (577 bp); and P4, −514 to +48 (563 bp); and *BrAGPS1b* P1, −2,000 to −1,270 (731 bp); P2, −1,175 to −389 (787 bp); and P3, −458 to +436 (894 bp). The motif distributions were used to design overlapping promoter fragments for Y1H bait construction.

The designed fragments were amplified and inserted into the pAbAi bait vector, and representative products were examined by agarose-gel electrophoresis (**Figure 3**).

**Figure 3:**
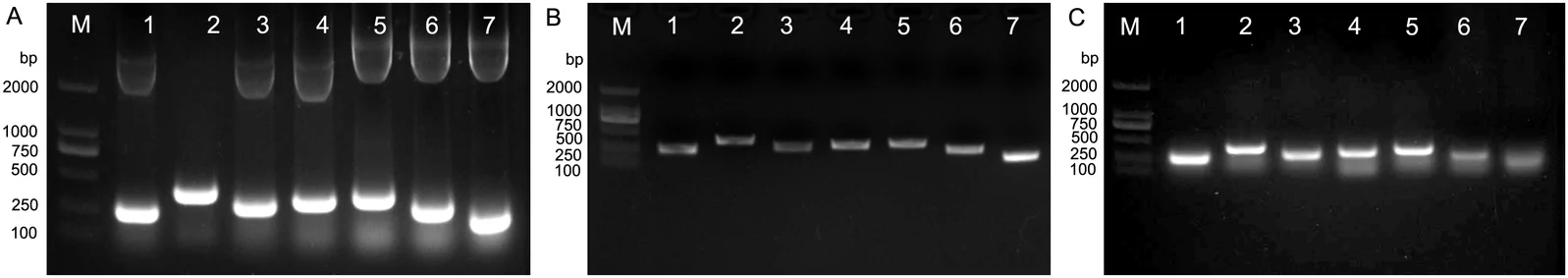
Agarose-gel verification of *BrAGPS1* promoter fragments used for bait-vector construction. M, DNA size marker; numbered lanes, amplified promoter fragments. **(A)** Using specific primers carrying KpnI and XhoI restriction sites, we amplified *BrAGPS1a* into four fragments (*BrAGPS1a*-1:244 bp, Lane 1; *BrAGPS1a*-2:380 bp, Lane 2; *BrAGPS1a*-3:282 bp, Lane 3; *BrAGPS1a*-4:320 bp, Lane 4) and *BrAGPS1b* into three fragments (*BrAGPS1b*-1:350 bp, Lane 5; *BrAGPS1b*-2:260 bp, Lane 6; *BrAGPS1b*-3:170 bp, Lane 7). **(B)** Electrophoretic detection of gel-extracted DNA fragments from panel **(A)**. **(C)** PCR screening of single colonies derived from *E. coli* DH5α transformed with the DNA products in panel **(B)**. M, DNA molecular weight marker (sizes in bp are indicated on the left).

### 3.3 Construction of Y1HGold bait yeast strains, self-activation testing, and library screening

The verified bait plasmids were linearized using BstBI and transformed into the Y1HGold yeast strain to facilitate integration of target promoter fragments into the yeast genome. Transformants with correctly integrated bait constructs were able to grow on SD/−Ura selective medium. Single colonies were randomly picked from SD/−Ura plates for each of the six recombinant constructs and validated by colony PCR using a combination of pAbAi universal primers and promoter-specific primers. Agarose gel electrophoresis confirmed that all PCR products matched the expected fragment sizes, verifying successful generation of the corresponding bait yeast strains.

To determine the minimum AbA concentration required to eliminate self-activation of each bait strain, the six bait strains and the positive control strain (p53-AbAi) were spot-inoculated on SD/−Ura plates supplemented with 0, 100, 150, 200, and 400 ng·mL^-1^ AbA. Consistent with expected results, the p53-AbAi control strain grew vigorously on AbA-free SD/−Ura medium but exhibited no growth in the presence of 100 ng·mL^-1^ AbA. Five bait strains, including pAbAi-BrAGPS1a-1, pAbAi-BrAGPS1a-3, pAbAi-BrAGPS1a-4, pAbAi-BrAGPS1b-1, and pAbAi-BrAGPS1b-3, displayed normal growth on AbA-free SD/−Ura medium and completely failed to grow at 100 ng·mL^-1^ AbA, indicating the absence of endogenous self-activation at this concentration. In contrast, the pAbAi-BrAGPS1a-2 strain still produced a small number of colonies on SD/−Ura medium containing 200 ng·mL^-1^ AbA, whereas no colony growth was observed at 400 ng·mL^-1^ AbA (**Figure 4**). Thus, the auto-activation threshold (minimum inhibitory AbA concentration) was 100 ng·mL^-1^ for pAbAi-BrAGPS1a-1, pAbAi-BrAGPS1a-3, pAbAi-BrAGPS1a-4, pAbAi-BrAGPS1b-1, and pAbAi-BrAGPS1b-3, and 400 ng·mL^-1^ for pAbAi-BrAGPS1a-2. An AbA concentration of 100 ng·mL^-1^ was adopted for library screening for the five self-activation-free bait strains, while 400 ng·mL^-1^ AbA was used for subsequent screening with the pAbAi-BrAGPS1a-2 bait strain.

**Figure 4:**
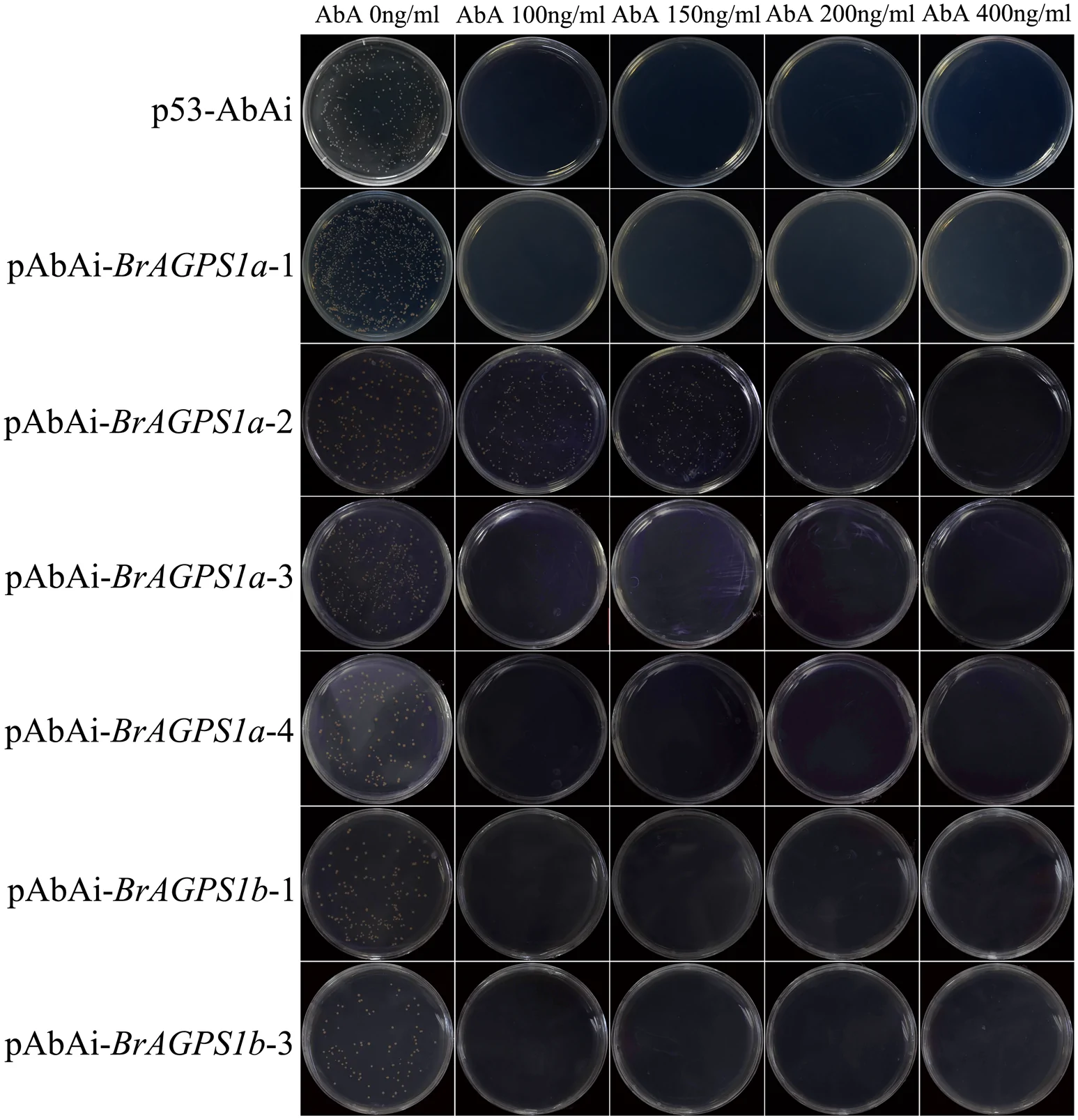
Self-activation testing of integrated *BrAGPS1* promoter bait strains on SD/−Ura medium containing increasing aureobasidin A (AbA) concentrations. Rows correspond to individual bait constructs and columns to the indicated AbA concentrations; the lowest concentration that suppressed bait autoactivation was used for subsequent screening. Positive and negative controls are described in the Methods.

Using the optimized AbA concentrations, Y1H library screening was performed to recover candidate cDNA inserts associated with the tested *BrAGPS1* promoter fragments (**Supplementary Figure S1**).

The pAbAi-*BrAGPS1a*-4 and pAbAi-*BrAGPS1b*-1 bait strains were selected for large-scale library screening. For pAbAi-*BrAGPS1a*-4, transformed yeast cells were plated onto SD/−Leu/AbA 100 ng·mL^-1^ plates (150-mm diameter) (**Supplementary Figures S1A, B**). Similarly, for pAbAi-*BrAGPS1b*-1, transformed cells were spread on another SD/−Leu/AbA 100 ng·mL^-1^ plates (**Supplementary Figures S1C, D**). Plates were incubated upside-down at 30 °C for 3–5 days. Colonies with diameters >1 mm were picked and restreaked onto fresh SD/−Leu/AbA 100 ng·mL^-1^ plates (90-mm diameter) for purification (**Supplementary Figures S1E, F**).

Library screening yielded 1,100 and 480 colonies for the *BrAGPS1a-4* and *BrAGPS1b-1* baits, respectively. Transformation efficiencies were approximately 1.2 × 10^6^ and 5.3 × 10^5^ CFU μg^-1^ DNA. Because 10 μg of library plasmid was used per transformation, the estimated total transformants were 1.2 × 10^7^ and 5.3 × 10^6^, respectively; both values exceeded the nominal library size of 1 × 10^6^ clones. We sequenced 56 single-band products from the *BrAGPS1a* screen and 31 from the *BrAGPS1b* screen. Within-screen deduplication yielded 54 and 29 unique sequences, respectively, and cross-bait deduplication yielded 76 nonredundant sequences in total. The size-based preselection described in the Methods may have excluded distinct inserts with similar amplicon sizes.

### 3.4 Functional annotation and classification of candidate proteins

Sequences were queried against NCBI and the *Brassica rapa* database BRAD by BLAST (Chen et al., 2022) and classified using database annotations, conserved domains, and published literature (**Supplementary Table S1**). Of 76 nonredundant sequences, 74 had an assigned or predicted function and two encoded uncharacterized proteins. Fifteen were annotated as having transcriptional, DNA-binding, histone-binding, or chromatin-associated potential, including one bZIP protein, one 3×HMG-box protein, two Alfin1-like PHD-domain proteins, and 11 proteins with zinc-finger, histone-binding, or chromatin-associated domains. The other 61 included signaling proteins, transporters, metabolic enzymes, structural proteins, and chaperones. Recovery in a library Y1H screen does not establish direct binding: metabolic enzymes and other noncanonical hits may represent false positives or uncharacterized promoter-associated candidates and require one-to-one back-assays and orthogonal validation.

Among the Y1H-derived genes, 28 met the differential-expression criteria at one or more sampled time points. One gene was differentially expressed at all four time points, and the largest time-specific set comprised nine genes detected only at 5 wpi (**Figure 5A**).

**Figure 5:**
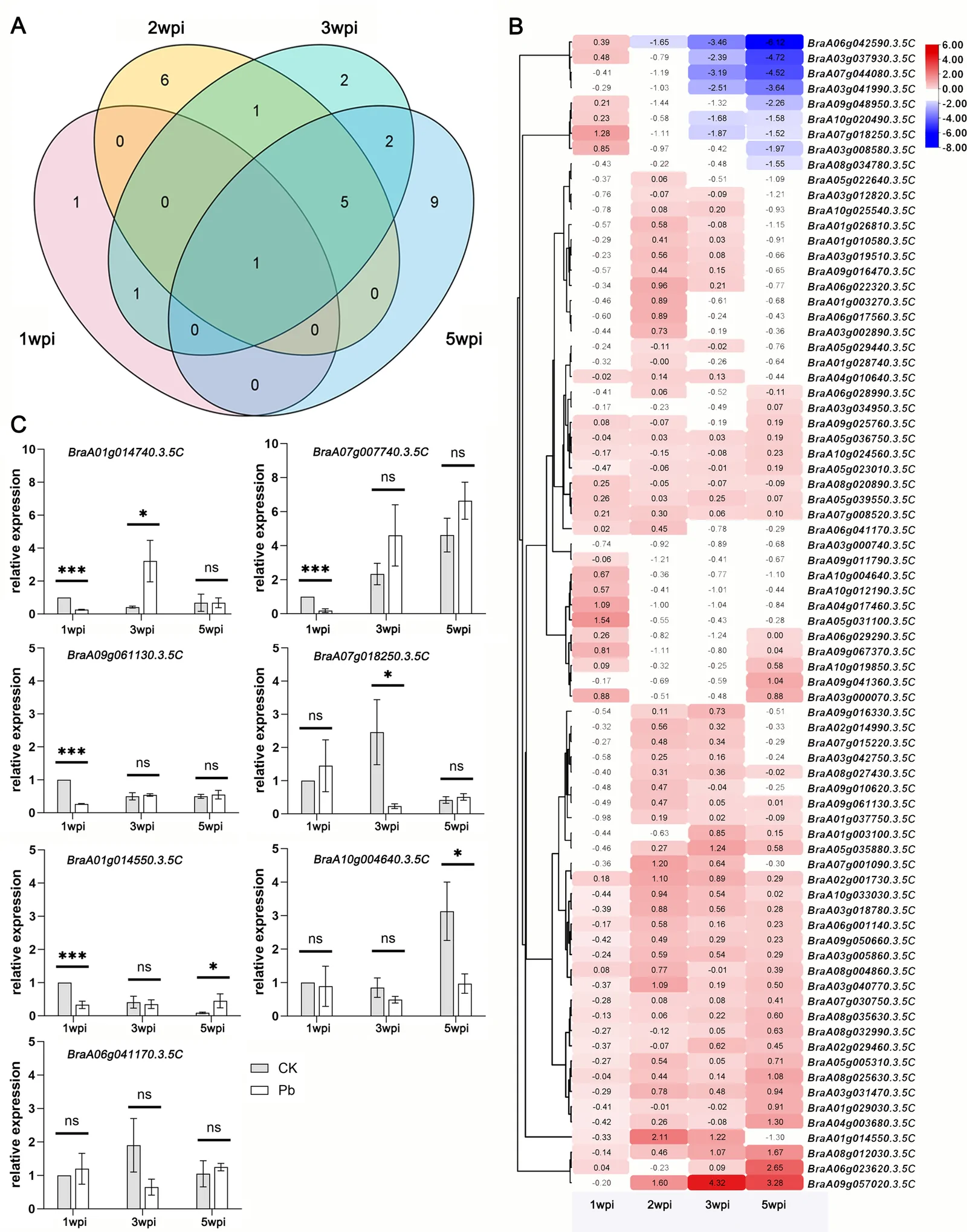
Temporal transcriptomic analysis of *P. brassicae* infection in Chinese cabbage roots. **(A)** Venn diagram of Y1H-derived genes differentially expressed between mock- and *P. brassicae*-inoculated roots at 1, 2, 3, and 5 wpi (Illumina NovaSeq 6000; DESeq2 analysis of raw counts; |log_2_FC| ≥ 1 and Benjamini–Hochberg-adjusted p ≤ 0.05). **(B)** Heatmap and hierarchical clustering of 76 genes identified by yeast one-hybrid screening, illustrating their temporal expression patterns at 1,2,3 and 5 wpi. **(C)** Quantitative real-time PCR (qRT-PCR) expression at 1, 3, and 5 wpi, normalized to *BrActin*. Bars show mean ± SD for three biological replicates; technical replicates were averaged before analysis. Inoculated and mock-control samples were compared within each time point by two-sided Student’s t-test: ns, not significant; *p< 0.05; **p< 0.01; ***p< 0.001.

Five genes were selected from the Y1H-derived candidates for qRT-PCR on the basis of their regulatory or signaling annotations together with infection-responsive RNA-seq profiles. The selected set represented a calcium-dependent protein kinase (*BraA07g018250.3.5C*), a bZIP transcription factor (*BraA10g004640.3.5C*), a 3×HMG-box protein (*BraA01g014550.3.5C*), and two Alfin1-like PHD-domain proteins (*BraA06g041170.3.5C* and *BraA09g061130.3.5C*). This prioritization did not establish sustained differential expression for every gene or direct interaction with an individual cis-element.

Across the infection time course, *BrAGPS1a* showed an early, transient increase at 1 wpi, whereas *BrAGPS1b* increased at 1 and 3 wpi but did not differ significantly from the mock-treated control at 5 wpi. The five prioritized candidates exhibited distinct temporal expression patterns. *BraA09g061130.3.5C* and *BraA01g014550.3.5C* showed lower transcript abundance at 1 wpi, whereas *BraA07g018250.3.5C* showed lower transcript abundance at 3 wpi. At 5 wpi, *BraA01g014550.3.5C* showed higher transcript abundance, whereas *BraA10g004640.3.5C* showed lower transcript abundance. *BraA06g041170.3.5C* did not differ significantly from the mock-treated control at any of the examined time points (**Figure 5C**).

Together, the Y1H library screening, functional annotation, RNA-seq, and qRT-PCR analyses prioritized five candidates for direct follow-up. **Figure 6** summarizes these candidates as four functional nodes: one calcium-dependent protein kinase, one bZIP transcription factor, one 3×HMG-box protein, and two Alfin1-like PHD-domain proteins. The working model places these candidate classes upstream of *BrAGPS1a/b* expression and outlines a possible relationship among *BrAGPS1a/b* expression, starch accumulation, glucose availability, and intracellular pathogen proliferation. The candidate-to-*BrAGPS1a/b* and *BrAGPS1a/b*-to-starch connectors are shown as dashed arrows because these relationships remain hypothetical. The solid arrow from starch accumulation to glucose represents starch degradation as a general metabolic process, whereas the dashed glucose-to-pathogen connector denotes the untested possibility that glucose released from starch may contribute to intracellular pathogen proliferation. The model does not demonstrate direct promoter binding, regulatory direction, carbon transfer to the pathogen, or a causal effect on pathogen proliferation.

**Figure 6:**
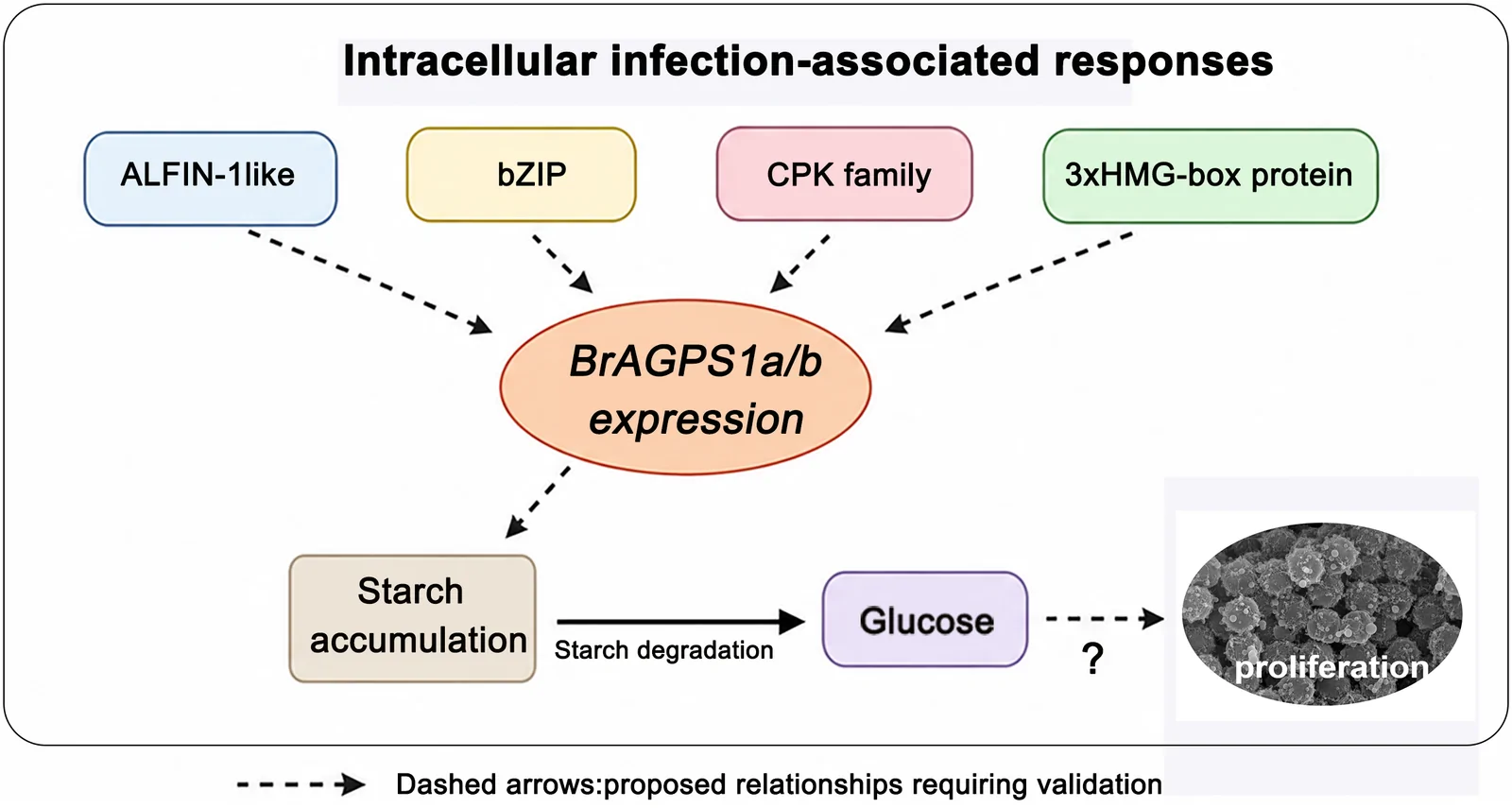
Working hypothesis for *BrAGPS1*-associated regulation and starch/carbon responses during intracellular *Plasmodiophora brassicae* infection in Chinese cabbage. The four upstream functional nodes represent five prioritized candidates: one CPK, one bZIP protein, one 3×HMG-box protein, and two Alfin1-like PHD-domain proteins. These candidate classes are positioned upstream of *BrAGPS1a/b* expression, which is provisionally connected to starch accumulation. The solid arrow from starch accumulation to glucose represents starch degradation, whereas the dashed connector between glucose and intracellular *P. brassicae* proliferation indicates an untested possibility. Dashed arrows denote proposed relationships that require independent experimental validation. The model does not establish direct promoter binding, regulatory direction, carbon transfer, or a causal effect on pathogen proliferation.

## 4 Discussion

### 4.1 Current status of clubroot resistance research and the underexplored role of host carbon metabolism regulation

Clubroot disease, caused by the obligate biotrophic pathogen *P. brassicae*, remains one of the most devastating soil-borne diseases limiting the production of Chinese cabbage (*Brassica rapa* var. *pekinensis*) and other cruciferous crops worldwide. Over the past decades, significant progress has been made in dissecting the genetic basis of clubroot resistance, with the cloning and functional validation of multiple dominant resistance loci including *CRa* (Ueno et al., 2012), *Crr1a* (Hatakeyama et al., 2013), *RPB1* (Ochoa et al., 2023), and *Crr5* (Yang et al., 2025), as well as the key susceptibility gene *GSL5* (Wu et al., 2025). These findings have greatly advanced our understanding of the molecular mechanisms underlying host–pathogen interactions in the Brassica-*P. brassicae* pathosystem (Sedaghatkish et al., 2025).

Most clubroot studies have centered on immune receptors and classical defense pathways, whereas the transcriptional regulation of infection-associated carbon partitioning is less well characterized. Our previous work reported starch accumulation and altered expression of starch-metabolism genes in infected Chinese cabbage roots (Ma et al., 2022). Building on that observation, the present study focused on *BrAGPS1*, which encodes a small subunit of ADP-glucose pyrophosphorylase, to nominate upstream candidates for testing. The present experiments do not establish whether the starch response benefits the host, the pathogen, or both.

### 4.2 Cis-regulatory element analysis of *BrAGPS1* promoters

To nominate candidate promoter-associated factors for *BrAGPS1* during *P. brassicae* infection, we cloned upstream regions of the two *BrAGPS1* homeologs, *BrAGPS1a* and *BrAGPS1b*, from ‘Chiifu-401-42’ and performed cis-regulatory motif prediction. Both sequences contained commonly predicted core promoter motifs (TATA-box and CAAT-box) and additional putative motifs associated with hormone, stress, and light responses. These sequence predictions provide hypotheses for testing and do not demonstrate motif function or transcriptional regulation in infected roots.

The predicted motifs include sequences associated in other systems with responses to abscisic acid, anaerobiosis, low temperature, gibberellin, salicylic acid, methyl jasmonate, light, and MYB/MYC-family factors. This motif collection generates hypotheses about possible inputs to *BrAGPS1* expression; prediction from sequence alone does not demonstrate that a motif is functional in infected Chinese cabbage roots.

Predicted MYB- and MYC-recognition sites provided one rationale for screening the promoters. However, the Y1H design used multi-element fragments and cannot link an individual recovered protein to a particular predicted site. Site-directed promoter mutagenesis and direct binding assays will be required to connect a candidate to a defined cis-element.

Library Y1H is useful for nominating proteins associated with a bait DNA sequence, but it is also prone to autoactivation, promiscuous reporter activation, and other false positives. The present screen therefore does not establish that noncanonical hits were recruited indirectly through protein complexes, nor does it assign a hit to a particular predicted motif. Metabolic enzymes, kinases, chaperones, and other unexpected hits should be retained only as preliminary candidates until they reproduce in one-to-one Y1H back-assays and are supported by direct binding or transactivation assays.

### 4.3 Correlation between differential expression dynamics and *P. brassicae* infection process

The number and identity of differentially expressed Y1H-derived genes varied across the infection time course. Because these samples represent mixtures of host cell types and pathogen developmental stages, the patterns cannot by themselves distinguish host defense, pathogen manipulation, or changes in tissue composition.

Early in the *P. brassicae* life cycle, primary zoospores infect root hairs and develop into primary plasmodia; subsequent secondary zoospores infect cortical cells. At 1 wpi, relatively few Y1H-derived genes met the differential-expression threshold, consistent with a limited or spatially restricted response in the sampled whole-root tissue.

During the 2–3-wpi interval, secondary plasmodia proliferate in cortical tissue and host cell division and enlargement become prominent (Tanaka et al., 2006). The increase in differentially expressed candidates at these time points is compatible with intensified host–pathogen interaction, but the data do not identify whether individual changes are initiated by host defense or pathogen activity.

By 5 wpi, enlarged clubs contain developing or mature resting spores and visible disease symptoms. The larger number of time-specific and downregulated candidates at this stage may reflect late infection, loss of normal root functions, or altered cell-type proportions. These alternatives require cell-resolved or genetic experiments to distinguish.

### 4.4 Functional value and research significance of core candidate genes

Integrating the Y1H-library hits with temporal expression data prioritized five genes for further testing. Their recovery with the screened promoter fragments and their infection-responsive expression provide two independent lines of candidate-level evidence, but neither establishes a causal *BrAGPS1* regulatory role.

*BraA07g018250.3*.5C encodes a calcium-dependent protein kinase, and calcium-dependent protein kinases can relay immune-associated calcium signals to downstream targets (Boudsocq et al., 2010; Kudla et al., 2018; Monaghan et al., 2014). *BraA01g014550.3*.5C encodes a 3×HMG-box protein, a class associated with chromatin architecture (Bustin and Reeves, 1996; Launholt et al., 2006). Their presence among the Y1H-derived hits supports alternative, testable hypotheses: they could participate in a host response, be exploited during infection, or be unrelated false-positive hits. One-to-one binding and perturbation experiments are needed before either host defense or pathogen co-option can be inferred. *BraA10g004640.3*.5C was annotated as a basic leucine zipper (bZIP) protein and is included as a separate candidate requiring direct interaction and functional validation.

The two Alfin1-like PHD-domain genes, *BraA06g041170.3.5C* and *BraA09g061130.3.5C*, encode candidates from a protein family associated with H3K4me3 recognition, root development, and stress-responsive regulation (Sanchez and Zhou, 2011; Mozgova and Hennig, 2015). Their RNA-seq and qRT-PCR patterns were not uniformly concordant across time points. Downregulation could reflect a host attempt to constrain developmental reprogramming, pathogen-mediated suppression, or changing tissue composition. The present data do not distinguish these possibilities or demonstrate regulation of *BrAGPS1*.

Disrupting *BrAGPS1* could, in principle, reduce infection-associated starch accumulation and alter the carbon environment available during *P. brassicae* colonization. The opposite outcome is also possible if starch accumulation is primarily a host protective or buffering response. Starch granules occur in infected cortical cells (Ikegami et al., 1984; Ma et al., 2022), but the present study did not measure carbon flux to the pathogen or pathogen performance after *BrAGPS1* perturbation. Targeted knockout or knockdown, coupled with starch quantification and pathogen-biomass or resting-spore measurements, is required to test causality.

This study used the susceptible inbred line ‘Chiifu-401-42’. Presence of a locus in the Chiifu reference genome does not establish sequence conservation or equivalent regulation in resistant germplasm. Genotyping the candidate loci and comparing their expression and function across resistant and susceptible accessions will be necessary to determine whether any association extends beyond the genotype studied here.

### 4.5 Limitations of the study and future research directions

This study has several limitations. First, the library Y1H results are candidate-level: one-to-one Y1H back-assays, electrophoretic mobility shift assays, and promoter-transactivation experiments are needed to verify direct binding and regulatory direction. Second, sequencing only one strong representative among PCR products of indistinguishable apparent size may have under-sampled different inserts of similar length. Third, candidate functions were inferred from annotation and expression; genetic perturbation is required to test effects on *BrAGPS1* expression, starch accumulation, and pathogen performance. Fourth, bulk-root RNA-seq cannot separate cell-type composition from within-cell regulation. Finally, the work used one susceptible genotype; validation in resistant and susceptible backgrounds is needed before relevance to clubroot resistance can be assessed.

## 5 Conclusions

In conclusion, analysis of the *BrAGPS1a* and *BrAGPS1b* upstream regions, Y1H library screening, and temporal expression profiling produced a set of 76 nonredundant candidate promoter-associated proteins. Fifteen had annotations consistent with transcriptional, DNA-binding, histone-binding, or chromatin-associated potential, and 28 Y1H-derived genes were differentially expressed at one or more infection time points. Five candidates were prioritized for follow-up on the basis of annotation and expression responsiveness. qRT-PCR documented distinct temporal expression patterns but did not validate binding or regulatory function. The study therefore provides hypotheses and experimental targets; direct binding, transactivation, genetic, and pathogen-response assays are required before a *BrAGPS1* regulatory mechanism or resistance role can be concluded.

## Data Availability

The datasets presented in this study can be found in online repositories. The names of the repository/repositories and accession number(s) can be found in the article/**Supplementary Material**.

## Supplementary material

Supplementary Figure 1 Yeast one-hybrid library screening plates. **(A, B)** Representative SD/−Leu/AbA (100 ng mL^-1^) plates from the *BrAGPS1a*-4 screen; **(C, D)** representative plates from the *BrAGPS1b*-1 screen; **(E, F)** purification plates after restreaking individual colonies. RNA-seq data are associated with NCBI BioProject PRJNA1516672.

Supplementary Table 1 Y1H-derived candidate genes and annotations.

Supplementary Table 2 primer sequences used in this study.
